# Supplementary material for: Violence Exposure and Mental Health Problems Among School-Aged Children in a South African Birth Cohort
Source: medRxiv. 2026 Apr 22:2026.04.20.26351289. Preprint. [Version 1] doi: 10.64898/2026.04.20.26351289 (PMC13131721; doi:10.64898/2026.04.20.26351289)
Supplement: Supplement 1 [file media-1.pdf]

Violence Exposure and Mental Health Problems Among School-Aged Children in a South African Birth Cohort

Supplementary Appendices

Table of Contents

Appendix 1. Linearity of polyvictimisation 2

Appendix 2. Open Science Framework (OSF) pre-registration 3

Appendix 3. Missing data 4

Appendix 4. Complete case analyses 15

Appendix 5. Linear regression diagnostics 24

Appendix 6. Comparison of sociodemographic characteristics for children included versus excluded in the analysis sample 27

Appendix 7. Prevalence of each traumatic event at the 4.5- and 8-year follow-ups 28

Appendix 8. Psychiatric disorders 31

Appendix 9. Sex differences 32

References 34

Appendix 1. Linearity of polyvictimisation

Polyvictimisation was captured by coding the number of subscales in which a child had experienced a traumatic event, with a maximum score of 4 indicating that the child had been exposed to events across all four subscales. We used likelihood-ratio tests to confirm linearity in polyvictimisation-psychopathology associations by comparing models capturing polyvictimisation as a categorical exposure and models capturing polyvictimisation as a numeric (continuous) exposure. Results are presented in Appendix 1b. One likelihood-ratio test was significant (the unadjusted longitudinal model for 'any psychiatric disorder'; p=0.026). Given all other tests were non-significant (as well as the fully adjusted version of this model), we proceeded with capturing polyvictimisation as a numeric (continuous) exposure in all our models.

Appendix 1b. Likelihood-ratio tests examining the linearity of the relationship between polyvictimisation and psychopathology at age 8

	χ^2	p
**Unadjusted: Longitudinal (polyvictimisation at 4.5 years)**		
Total CBCL score	2.15	0.542
Internalizing CBCL score	3.40	0.335
Externalizing CBCL score	2.84	0.417
Any psychiatric disorder (MINI)	9.31	0.026
**Adjusted: Longitudinal (polyvictimisation at 4.5 years)**		
Total CBCL score	2.19	0.535
Internalizing CBCL score	1.91	0.591
Externalizing CBCL score	3.78	0.286
Any psychiatric disorder (MINI)	5.15	0.162
**Unadjusted: Cross-sectional (polyvictimisation at 8 years)**		
Total CBCL score	1.36	0.714
Internalizing CBCL score	2.12	0.548
Externalizing CBCL score	2.58	0.460
Any psychiatric disorder (MINI)	5.83	0.120
**Adjusted: Cross-sectional (polyvictimisation at 8 years)**		
Total CBCL score	1.17	0.759
Internalizing CBCL score	1.45	0.695
Externalizing CBCL score	1.96	0.580
Any psychiatric disorder (MINI)	4.87	0.181

Note. The degrees of freedom was 3 for all analyses. *Key:* CBCL=Child Behavior Checklist; MINI=Mini International Neuropsychiatric Interview.

Appendix 2. Open Science Framework (OSF) pre-registration

This study was pre-registered on the Open Science Framework on May 14th, 2025. We were unable to carry out some of our analysis plans. Firstly, alongside examining the association between violence exposure and 'any psychiatric disorder' (assessed using the Mini International Neuropsychiatric Interview for Children and Adolescents),[1] we planned to examine this association separately for internalizing disorders (meeting criteria for at least one of the following disorders: generalizing anxiety disorder, obsessive compulsive disorder, posttraumatic stress disorder, separation anxiety disorder, social anxiety disorder, specific phobia, and major depressive disorder) and externalizing disorders (meeting criteria for at least one of the following disorders: attention deficit hyperactivity disorder, conduct disorder, and oppositional defiant disorder). However, few children met criteria for any of the assessed psychiatric disorders (*n*=31 for internalizing disorders and *n*=67 for externalizing disorders), and we were therefore unable to examine these associations with sufficient statistical power. Secondly, we planned to estimate the proportion of mental health problems (using the Child Behaviour Checklist; CBCL)[2] and disorders (MINI-KID) at age 8 that could be explained by any violence exposure up to age 8 using population attributable fractions. We planned to binary code violence exposure to capture exposed versus unexposed children as well as to binary code CBCL *t*-scores to capture children with clinically significant elevations. We were unable to conduct these analyses, again due to low frequencies and insufficient statistical power. The majority of children (91.1%) were violence exposed at age 8, while in contrast few children had clinically significant elevations on the CBCL (*n*=47 for the total problems score, *n*=63 for the internalizing problems score, and *n*=63 for the externalizing problems score) or met criteria for a psychiatric disorder (*n*=91). We also made a minor change to our cross-sectional models. The variation in child age at the 8-year follow-up was larger than what we were expecting, with some children as old as 10.5 years when the measures were assessed for this wave. Prior to conducting any analyses, we decided to include child age at the 8-year Child Exposure to Community Violence Checklist (CECV) assessment as a confounder in our cross-sectional models, given that we hypothesised that child age could be associated with both violence exposure scores and mental health problems. Longitudinal models were carried out as planned.

Appendix 3. Missing data

Appendix 3a. Patterns and approach

Missingness for violence exposure was 23.5% at 4.5 years and 14.2% at 8 years. Missingness for age 8 psychopathology was 16.1% for the CBCL and 15.5% for the MINI-KID. A summary of missing data for all analysis variables, confounders, and auxiliary variables used in the imputation process is presented in Appendix 3b.

A comparison of children with complete data and children with missing data for violence exposure at ages 4.5 and 8 years and/or psychopathology at age 8 are presented in Appendices 3c and 3d. Complete case analyses are valid if the analysis model outcome is unrelated to missingness, conditional on the analysis model covariates.[3] While the association between the outcome and missingness cannot be examined using observed data, we are able to examine whether this assumption is violated because of a confounder that is associated with both missingness and the analysis model outcome. Findings for analyses investigating the association between our confounders and model outcomes are presented in Appendix 3e. Child ethnicity, maternal alcohol use during pregnancy, and maternal smoking during pregnancy were all associated with at least one outcome variable and with missingness. Given that these variables were also considered confounders (decided a priori based on a directed acyclic graph), we adjusted our models for these variables to avoid bias. Child age at the 8-year CECV assessment was also significantly associated with at least one model outcome and missingness in the cross-sectional analyses and was therefore also adjusted for in our cross-sectional analyses. Additionally, the total violence exposure scores at ages 3.5 and 4.5 years were also significantly associated with our outcome variables and with missingness. Given that our violence exposure scores reflect lifetime exposure, we were unable to condition on these variables. However, violence scores across all available timepoints (3.5, 4.5, 6, and 8 years), with different patterns of missingness, were included in the imputation models as auxiliary variables to make the missing at random assumption more plausible. Finally, the CBCL total problems score at 2 years was also significantly associated with our outcome variables and with missingness in our longitudinal analyses, and this was also used as an auxiliary variable in our imputation analyses.

We used multivariate imputation by chained equations with 50 imputed datasets to address missing data (N=974).[4] We used Stata Version 18. Auxiliary variables included: total mental health problem scores assessed using the CBCL at 2 years, 5 years, and 6.5 years and CECV

subscale scores at ages 3.5 and 6 years. Two imputation models were used. The first model imputed the CECV subscale scores at 4.5 and 8 years with all outcome, confounder, and auxiliary variables. The total CECV score was then calculated after imputation. The second model was necessary due to multicollinearity when the subscale and the polyvictimisation scores were included in the same model. We therefore passively imputed the polyvictimisation scores at 4.5 and 8 years from the subscales. Due to already capturing the subscale-outcome associations in the first imputation model, we were able to remove the individual subscales from the imputation equations for the outcomes and confounders, while including the polyvictimisation variables, which addressed multicollinearity. Convergence checks prior to imputation revealed that the community victimization score at 4.5 years perfectly predicted the 'any psychiatric disorder' variable in males; the 'augment' command was therefore added to the imputation equation for the 'any disorder' variable. All binary variables were imputed using logistic regression and all continuous variables were imputed using predictive mean matching. Variables were also imputed by sex, enabling the examination of sex differences. All Monte Carlo errors for effect estimates were less than 10% of its standard error, suggesting 50 imputed datasets was sufficient.[5] Stata do-files, which detail our imputation model inclusions and exclusions, are available open access on GitHub (https://github.com/megan-l-bailey/DCHS-Violence-MH).

Appendix 3b. Summary of missing data (*N*=974)

Variable	Description	Missing values	Range	Mean	SD	Skewness	Kurtosis
**Main Analysis Variables**							
`totcbcl_8y`	CBCL total score at age 8 years	157 (16.1%)	24-80	45.03	11.82	0.626	0.011
`intcbcl_8y`	CBCL internalizing score at age 8 years	157 (16.1%)	33-83	43.96	9.50	<0.001	0.911
`extcbcl_8y`	CBCL externalizing score at age 8	157 (16.1%)	33-77	48.14	10.33	<0.001	0.003
`anydis`	Any MINI-KID disorder at age 8 years	151 (15.5%)	0/1	-	-	-	-
`totalcecv_54m`	CECV total score at age 4.5 years	229 (23.5%)	0-27	3.55	3.99	<0.001	<0.001
`witcom_54m`	CECV witnessed community violence at age 4.5	229 (23.5%)	0-22	2.35	2.69	<0.001	<0.001
`comvic_54m`	CECV community victimization at age 4.5	229 (23.5%)	0-4	0.15	0.52	<0.001	<0.001
`witdom_54m`	CECV witnessed domestic violence at age 4.5	229 (23.5%)	0-6	0.46	0.96	<0.001	<0.001
`domvic_54m`	CECV domestic victimization at age 4.5	229 (23.5%)	0-11	0.59	1.46	<0.001	<0.001
`polyvic_54m`	CECV polyvictimisation at age 4.5	229 (23.5%)	0-4	1.22	0.99	<0.001	0.817
`totalcecv_8y`	CECV total score at age 8 years	138 (14.2%)	0-41	5.93	4.99	<0.001	<0.001
`witcom_8y`	CECV witnessed community violence at age 8	138 (14.2%)	0-16	2.95	2.91	<0.001	<0.001
`comvic_8y`	CECV community victimization at age 8	138 (14.2%)	0-9	0.58	1.23	<0.001	<0.001
`witdom_8y`	CECV witnessed domestic violence at age 8	138 (14.2%)	0-5	0.45	0.90	<0.001	<0.001
`domvic_8y`	CECV domestic victimization at age 8	138 (14.2%)	0-20	1.94	2.56	<0.001	<0.001
`polyvic_8y`	CECV polyvictimisation at age 8	138 (14.2%)	0-4	1.81	1.04	0.022	<0.001
**Confounders**							
`sex`	Child sex	0	0/1	-	-	-	-
`ethnicity`	Child ethnicity	0	0/1	-	-	-	-
`income`	Monthly family income at birth	0	0/1/2	-	-	-	-
`education`	Maternal education at birth	0	0/1	-	-	-	-

work	Maternal work status at birth	0	0/1	-	-	-	-
marital_status	Maternal marital status at birth	0	0/1	-	-	-	-
malcohol	Maternal prenatal alcohol consumption	41 (4.2%)	0/1	-	-	-	-
msmoking	Maternal prenatal smoking	0	0/1	-	-	-	-
chiv_birth	Child HIV exposure status at birth	0	0/1	-	-	-	-
mdepression	Maternal antenatal depression (EPDS score)	111 (11.4%)	0-26	9.51	5.26	<0.001	0.103
age_8y	Child age at the 8-year CECV assessment	138 (14.2%)	7.77-10.58	8.46	0.50	<0.001	<0.001
totcbcl_42m	CBCL total score at 3.5 years	77 (7.9%)	28-89	39.43	9.95	<0.001	0.002
**Auxiliary Variables**							
totcbcl_24m	CBCL total score at age 2 years	342 (35.1%)	28-85	45.13	11.88	<0.001	0.385
totcbcl_60m	CBCL total score at age 5 years	123 (12.6%)	28-86	41.44	12.34	<0.001	0.135
totcbcl_78m	CBCL total score at age 6.5 years	147 (15.1%)	24-74	42.38	10.29	0.010	0.733
totalcecv_42m	CECV total score at age 4.5 years	450 (46.2%)	0-27	3.43	3.99	<0.001	<0.001
witcom_42m	CECV witnessed community violence at age 3.5	450 (46.2%)	0-18	2.15	2.64	<0.001	<0.001
comvic_42m	CECV community victimization at age 3.5	450 (46.2%)	0-6	0.14	0.56	<0.001	<0.001
witdom_42m	CECV witnessed domestic violence at age 3.5	450 (46.2%)	0-9	0.54	1.03	<0.001	<0.001
domvic_42m	CECV domestic victimization at age 3.5	450 (46.2%)	0-10	0.60	1.36	<0.001	<0.001
polyvic_42m	CECV polyvictimisation at age 3.5	450 (46.2%)	0-4	1.21	1.03	<0.001	0.129
totalcecv_6y	CECV total score at age 6 years	439 (45.1%)	0-46	3.90	4.68	<0.001	<0.001
witcom_6y	CECV witnessed community violence at age 6	439 (45.1%)	0-16	2.40	2.74	<0.001	<0.001
comvic_6y	CECV community victimization at age 6	439 (45.1%)	0-6	0.22	0.74	<0.001	<0.001
witdom_6y	CECV witnessed domestic violence at age 6	439 (45.1%)	0-8	0.40	1.01	<0.001	<0.001
domvic_6y	CECV domestic victimization at age 6	439 (45.1%)	0-20	0.87	1.96	<0.001	<0.001
polyvic_6y	CECV polyvictimisation at age 6	439 (45.1%)	0-4	1.27	1.06	<0.001	0.472

Key: CBCL=Child Behavior Checklist; CECV=Child Exposure to Community Violence Checklist; EPDS=Edinburgh Postnatal Depression Scale; MINI-KID=Mini International Neuropsychiatric Interview for Children and Adolescents.

Appendix 3c. Comparison of complete cases and those with missing information for violence exposure at age 4.5 years and mental health problems at age 8 years

**Variable**	**Proportion missingness**	**Complete Cases (*n*=545)**		**Missing Cases (*n*=429)**		**Association with missingness**	
	n(%)	*n*	(%) or M(SD)	*n*	(%) or M(SD)	OR (95% CI)	*p*-value
**Exposure**							
CBCL total score at 2 years	342 (35.1%)	376	46.09 (12.31)	256	43.70 (11.08)	0.98 (0.97, 1.00)	0.013
CECV total score at age 3.5 years	450 (46.2%)	349	3.80 (4.17)	175	2.69 (3.51)	0.92 (0.87, 0.97)	0.003
**Confounders**							
Female sex	0 (0%)	265	(48.6%)	214	(49.9%)	1.05 (0.82, 1.35)	0.696
Child ethnicity (Black African)[a]	0 (0%)	257	(47.2%)	273	(63.6%)	1.96 (1.51, 2.54)	<0.001
Child HIV exposure (exposed, uninfected)[b]	0 (0%)	110	(20.2%)	102	(23.8%)	1.23 (0.91, 1.67)	0.178
Monthly family income[c]	0 (0%)						
R1000-5000/m		269	(49.4%)	242	(56.4%)	1.33 (1.01, 1.75)	0.044
>R5000/m		72	(13.2%)	49	(11.4%)	1.01 (0.66, 1.53)	0.978
Maternal education (completed secondary/any tertiary)[d]	0 (0%)	196	(36.0%)	168	(39.2%)	1.15 (0.88, 1.49)	0.306
Maternal employment status (working)[e]	0 (0%)	133	(24.4%)	122	(28.4%)	1.23 (0.92, 1.64)	0.155
Maternal marital status (married/cohabiting)[f]	0 (0%)	219	(40.2%)	170	(39.6%)	0.98 (0.75, 1.27)	0.860
Maternal prenatal alcohol use (yes)	41 (4.2%)	93	(17.1%)	27	(7.0%)	0.36 (0.23, 0.57)	<0.001
Maternal prenatal smoking (yes)	0 (0%)	198	(36.3%)	89	(20.8%)	0.46 (0.34, 0.61)	<0.001
Maternal antenatal depression	111 (11.4%)	545	9.45 (5.32)	318	9.62 (5.16)	1.01 (0.98, 1.03)	0.660
CBCL total score at 3.5 years	77 (7.9%)	545	39.84 (10.35)	352	38.79 (9.28)	0.99 (0.98, 1.00)	0.124

Note. [a]Reference group is mixed ancestry. [b]Reference group is HIV unexposed. [c]Reference group is <R1000/m. [d]Reference group is primary/some secondary. [e]Reference group is not working. [f]Reference group is single. *Key:* CBCL=Child Behavior Checklist; CECV=Child Exposure to Community Violence Checklist; CI=confidence interval; M=mean; OR=odds ratio; SD=standard deviation.

Appendix 3d. Comparison of complete cases and those with missing information for violence exposure and mental health problems at age 8 years

**Variable**	**Proportion missingness**	**Complete Cases (*n*=659)**		**Missing Cases (*n*=315)**		**Association with missingness**	
	n(%)	*n*	(%) or M(SD)	*n*	(%) or M(SD)	OR (95% CI)	*p*-value
**Exposure**							
CBCL total score at age 2 years	342 (35.1%)	447	45.62 (12.09)	185	43.94 (11.28)	0.99 (0.97, 1.00)	0.106
CBCL total score at age 5 years	123 (12.6%)	604	41.48 (12.27)	247	41.36 (12.52)	1.00 (0.99, 1.01)	0.905
CBCL total score at age 6.5 years	147 (15.1%)	565	42.48 (10.22)	262	42.15 (10.45)	1.00 (0.98, 1.01)	0.662
CECV total score at age 3.5 years	450 (46.2%)	397	3.67 (4.07)	127	2.68 (3.66)	0.93 (0.87, 0.99)	0.016
CECV total score at age 4.5 years	229 (23.5%)	545	3.81 (4.11)	200	2.87 (3.54)	0.93 (0.89, 0.98)	0.005
CECV total score at age 6 years	439 (45.1%)	417	4.01 (4.88)	118	3.50 (3.86)	0.97 (0.93, 1.02)	0.297
**Confounders**							
Female sex	0 (0%)	323	(49.0%)	156	(49.5%)	1.02 (0.78, 1.34)	0.882
Child ethnicity (Black African)[a]	0 (0%)	320	(48.6%)	210	(66.7%)	2.12 (1.60, 2.80)	<0.001
Child HIV exposure (exposed, uninfected)[b]	0 (0%)	129	(19.6%)	83	(26.4%)	1.47 (1.07, 2.02)	0.017
Monthly family income[c]	0 (0%)						
R1000-5000/m		327	(49.6%)	184	(58.4%)	1.38 (1.03, 1.86)	0.032
>R5000/m		89	(13.5%)	32	(10.2%)	0.88 (0.55, 1.41)	0.600
Maternal education (completed secondary/any tertiary)[d]	0 (0%)	242	(36.7%)	122	(38.7%)	1.09 (0.83, 1.44)	0.545
Maternal employment status (working)[e]	0 (0%)	163	(24.7%)	92	(29.2%)	1.26 (0.93, 1.70)	0.138
Maternal marital status (married/cohabiting)[f]	0 (0%)	263	(39.9%)	126	(40.0%)	1.00 (0.76, 1.32)	0.978
Maternal prenatal alcohol use (yes)	41 (4.2%)	99	(15.0%)	21	(7.7%)	0.47 (0.29, 0.77)	0.003
Maternal prenatal smoking (yes)	0 (0%)	225	(34.1%)	62	(19.7%)	0.47 (0.34, 0.65)	<0.001

Maternal antenatal depression	111 (11.4%)	659	9.42 (5.28)	204	9.83 (5.18)	1.01 (0.99, 1.05)	0.328
Child age at the 8-year CECV assessment	138 (14.2%)	659	8.42 (0.43)	177	8.61 (0.69)	2.05 (1.49, 2.81)	<0.001
CBCL total score at 3.5 years	77 (7.9%)	659	39.70 (10.23)	238	38.66 (9.10)	0.99 (0.97, 1.00)	0.164

Note. [a]Reference group is mixed ancestry. [b]Reference group is HIV unexposed. [c]Reference group is <R1000/m. [d]Reference group is primary/some secondary. [e]Reference group is not working. [f]Reference group is single. *Key:* CBCL=Child Behavior Checklist; CECV=Child Exposure to Community Violence Checklist; CI=confidence interval; M=mean; OR=odds ratio; SD=standard deviation.

Appendix 3e. Associations between confounders and model outcomes at age 8 years

	**CBCL Total Score**		**CBCL Internalizing Score**		**CBCL Externalizing Score**		**Any Psychiatric Disorder**	
	B (95% CI)	*p*	B (95% CI)	*p*	B (95% CI)	*p*	OR (95% CI)	*p*
**Confounders**								
Female sex	-3.17 (-4.97, -1.37)	0.001	-3.72 (-5.15, -2.30)	<0.001	-2.67 (-4.25, -1.08)	0.001	0.21 (0.11, 0.39)	<0.001
Child ethnicity (Black African)[a]	-2.03 (-3.84, -0.22)	0.028	-2.95 (-4.39, -1.51)	<0.001	-1.87 (-3.46, -0.28)	0.022	1.00 (0.61, 1.64)	0.996
Child HIV exposure (exposed, uninfected)[B]	0.008 (-2.28, 2.30)	0.995	-0.75 (-2.59, 1.08)	0.420	-0.42 (-2.44, 1.59)	0.682	1.27 (0.71, 2.29)	0.424
Monthly family income (R1000-5000/m)[c]	0.65 (-1.33, 2.62)	0.520	0.38 (-1.20, 1.97)	0.634	0.65 (-1.09, 2.39)	0.460	0.72 (0.42, 1.23)	0.230
Monthly family income (>R5000/m) [c]	0.62 (-2.27, 3.51)	0.673	0.73 (-1.59, 3.04)	0.539	0.30 (-2.25, 2.84)	0.819	1.17 (0.58, 2.39)	0.660
Maternal education (completed secondary/any tertiary)[d]	-0.15 (-2.03, 1.73)	0.874	-1.04 (-2.55, 0.47)	0.176	-0.23 (-1.89, 1.42)	0.781	1.17 (0.71, 1.94)	0.532
Maternal employment status (working)[e]	0.18 (-1.93, 2.28)	0.869	-0.68 (-2.36, 1.01)	0.432	0.09 (-1.77, 1.94)	0.927	0.99 (0.56, 1.75)	0.973
Maternal marital status (married/cohabiting)[f]	1.72 (-0.13, 3.57)	0.068	1.58 (0.10, 3.07)	0.036	1.61 (-0.02, 3.24)	0.053	1.15 (0.70, 1.89)	0.583
Maternal prenatal alcohol use (yes)	3.36 (0.83, 5.88)	0.009	3.60 (1.58, 5.62)	<0.001	2.50 (0.27, 4.73)	0.028	1.23 (0.64, 2.35)	0.538
Maternal prenatal smoking (yes)	3.39 (1.49, 5.28)	<0.001	2.83 (1.31, 4.35)	<0.001	2.96 (1.29, 4.63)	0.001	1.17 (0.70, 1.94)	0.556
Maternal antenatal depression	0.29 (0.12, 0.46)	0.001	0.22 (0.09, 0.36)	0.001	0.24 (0.09, 0.39)	0.002	1.01 (0.97, 1.06)	0.587

Child age at the 8-year CECV assessment	0.58 (-1.54, 2.71)	0.591	1.73 (0.02, 3.43)	0.047	1.20 (-0.67, 3.07)	0.208	0.65 (0.36, 1.20)	0.169
CBCL total score at 3.5 years	0.16 (0.08, 0.25)	<0.001	0.13 (0.06, 0.20)	<0.001	0.13 (0.06, 0.21)	0.001	1.03 (1.01, 1.05)	0.008
**Auxiliary Variables**								
CBCL total score at age 2 years	0.22 (0.13, 0.31)	<0.001	0.15 (0.08, 0.22)	<0.001	0.19 (0.11, 0.27)	<0.001	1.01 (0.99, 1.04)	0.241
CBCL total score at age 5 years	0.20 (0.12, 0.28)	<0.001	0.11 (0.05, 0.18)	<0.001	0.18 (0.11, 0.25)	<0.001	1.02 (1.00, 1.04)	0.105
CBCL total score at age 6.5 years	0.34 (0.25, 0.43)	<0.001	0.18 (0.11, 0.26)	<0.001	0.32 (0.24, 0.40)	<0.001	1.06 (1.03, 1.09)	<0.001
CECV total score at age 3.5 years	0.73 (0.43, 1.02)	<0.001	0.66 (0.43, 0.90)	<0.001	0.67 (0.41, 0.93)	<0.001	1.09 (1.02, 1.16)	0.006
CECV total score at age 4.5 years	0.47 (0.23, 0.71)	<0.001	0.45 (0.26, 0.65)	<0.001	0.38 (0.17, 0.60)	<0.001	1.02 (0.96, 1.08)	0.536
CECV total score at age 6 years	0.51 (0.27, 0.74)	<0.001	0.45 (0.27, 0.63)	<0.001	0.41 (0.20, 0.62)	<0.001	1.04 (0.99, 1.10)	0.094

Note. For confounder associations, analyses are based on complete case data for the total violence exposure score at age 8, mental health problems at age 8, and confounders (*n*=659). For auxiliary variable associations, analyses are additionally based on complete data for each of the variables of interest (*n*=447 for CBCL total at 2 years; *n*=604 for CBCL total at 5 years; *n*=565 for CBCL total at 6.5 years; *n*=397 for CECV total at 3.5 years; *n*=545 for CECV total at 4.5 years; and *n*=417 for CECV total at 6 years). [a]Reference group is mixed ancestry. [b]Reference group is HIV unexposed. [c]Reference group is <R1000/m. [d]Reference group is primary/some secondary. [e]Reference group is not working. [f]Reference group is single. Key: B=unstandardized beta; CBCL=Child Behavior Checklist; CECV=Child Exposure to Community Violence Checklist; CI=confidence interval; OR=odds ratio.

Appendix 4. Complete case analyses

Appendix 4a. Sociodemographic characteristics, trauma exposure, and mental health outcome descriptives

	Complete Cases	*n* (%)	Mean (SD)	Range
**Sociodemographic characteristics**				
Child sex (female)	974	479 (49.2%)		
Child ethnicity (Black African)[a]	974	530 (54.4%)		
Child HIV exposure (exposed but uninfected)[b]	974	212 (21.8%)		
Monthly family income	974			
<R1000		342 (35.1%)		
R1000-5000		511 (52.5%)		
>R5000		121 (12.4%)		
Maternal education (completed secondary/any tertiary)[c]	974	364 (37.4%)		
Maternal employment status (working)[d]	974	255 (26.2%)		
Maternal marital status (married/cohabiting)[e]	974	389 (39.9%)		
Maternal prenatal alcohol use (yes)	933	120 (12.9%)		
Maternal prenatal tobacco use (yes)	974	287 (29.5%)		
Maternal antenatal depression	863		9.51 (5.26)	0-26
Child age at the 8-year CECV assessment	836		8.46 (0.50)	7.77-10.58
**Child trauma exposure – CECV scores at 4.5 years**				
Total	745	559 (75.0%)	3.55 (3.99)	0-27
Witnessed community violence	745	496 (66.6%)	2.35 (2.69)	0-22
Community victimization	745	67 (9.0%)	0.15 (0.52)	0-4
Witnessed domestic violence	745	178 (23.9%)	0.46 (0.96)	0-6
Domestic victimization	745	168 (22.6%)	0.59 (1.46)	0-11
Polyvictimisation	745		1.22 (0.99)	0-4

0		186 (25.0%)		
1		309 (41.5%)		
2		166 (22.3%)		
3		68 (9.1%)		
4		16 (2.2%)		
**Child trauma exposure – CECV scores at 8 years**				
Total	836	760 (90.9%)	5.93 (4.99)	0-41
Witnessed community violence	836	635 (76.0%)	2.95 (2.91)	0-16
Community victimization	836	230 (27.5%)	0.58 (1.23)	0-9
Witnessed domestic violence	836	204 (24.4%)	0.45 (0.90)	0-5
Domestic victimization	836	442 (52.9%)	1.94 (2.56)	0-20
Polyvictimisation	836		1.81 (1.04)	0-4
0		76 (9.1%)		
1		277 (33.1%)		
2		260 (31.1%)		
3		178 (21.3%)		
4		45 (5.4%)		
**Child mental health**				
CBCL total score at 3.5 years	897		39.43 (9.95)	28-89
CBCL total score at 8 years	817		45.03 (11.82)	24-80
CBCL internalizing score at 8 years	817		43.96 (9.50)	33-83
CBCL externalizing score at 8 years	817		48.14 (10.33)	33-77
Any psychiatric disorder at 8 years	823	91 (11.1%)		

Note. Full sample demographics and descriptive statistics (N=974). [a]Reference group is mixed ancestry. [b]Reference group is HIV unexposed. [c]Reference group is primary/some secondary. [d]Reference group is not working. [e]Reference group is single. *Key*: CBCL=Child Behavior Checklist; CECV=Child Exposure to Community Violence Checklist.

Appendix 4b. Cross-sectional associations between trauma exposure score at age 8 years and mental health problems at age 8 years

	**Total CBCL Score**			**Internalizing CBCL Score**			**Externalizing CBCL Score**		
	B (95% CI)	SE	*p*	B (95% CI)	SE	*p*	B (95% CI)	SE	*p*
**Unadjusted**									
Overall violence exposure	0.58 (0.39, 0.77)	0.10	<0.001	0.41 (0.23, 0.58)	0.09	<0.001	0.52 (0.35, 0.69)	0.09	<0.001
Witnessed community violence	0.61 (0.32, 0.89)	0.15	<0.001	0.56 (0.32, 0.80)	0.12	<0.001	0.55 (0.29, 0.82)	0.14	<0.001
Community victimization	1.02 (0.15, 1.90)	0.45	0.022	0.70 (0.01, 1.40)	0.35	0.048	0.98 (0.24, 1.73)	0.38	0.009
Witnessed domestic violence	1.81 (0.73, 2.89)	0.55	0.001	1.33 (0.43, 2.24)	0.46	0.004	1.49 (0.52, 2.46)	0.49	0.003
Domestic victimization	0.88 (0.52, 1.25)	0.18	<0.001	0.44 (0.11, 0.77)	0.17	0.010	0.79 (0.47, 1.11)	0.16	<0.001
Polyvictimisation	2.47 (1.62, 3.32)	0.43	<0.001	1.57 (0.87, 2.27)	0.36	<0.001	2.15 (1.41, 2.88)	0.37	<0.001
**Adjusted (confounders)**									
Overall violence exposure	0.48 (0.29, 0.67)	0.10	<0.001	0.29 (0.12, 0.45)	0.09	0.001	0.44 (0.27, 0.61)	0.09	<0.001
Witnessed community violence	0.47 (0.16, 0.78)	0.16	0.003	0.31 (0.05, 0.57)	0.13	0.019	0.42 (0.13, 0.70)	0.15	0.004
Community victimization	0.70 (-0.13, 1.53)	0.42	0.099	0.40 (-0.24, 1.03)	0.32	0.220	0.73 (0.01, 1.44)	0.36	0.047
Witnessed domestic violence	1.29 (0.21, 2.37)	0.55	0.019	0.83 (-0.03, 1.69)	0.44	0.059	1.06 (0.09, 2.02)	0.49	0.032
Domestic victimization	0.92 (0.56, 1.28)	0.18	<0.001	0.51 (0.18, 0.84)	0.17	0.003	0.85 (0.53, 1.17)	0.16	<0.001
Polyvictimisation	2.06 (1.19, 2.93)	0.44	<0.001	1.16 (0.46, 1.85)	0.35	0.001	1.83 (1.09, 2.58)	0.38	<0.001
**Adjusted (confounders and CBCL total score at age 3.5 years)**									
Overall violence exposure	0.44 (0.25, 0.63)	0.10	<0.001	0.25 (0.08, 0.42)	0.09	0.004	0.41 (0.24, 0.58)	0.09	<0.001
Witnessed community violence	0.39 (0.07, 0.71)	0.16	0.016	0.25 (-0.02, 0.51)	0.14	0.068	0.36 (0.07, 0.65)	0.15	0.016
Community victimization	0.61 (-0.22, 1.44)	0.42	0.151	0.33 (-0.31, 0.97)	0.33	0.315	0.66 (-0.06, 1.37)	0.36	0.071
Witnessed domestic violence	1.20 (0.12, 2.28)	0.55	0.029	0.76 (-0.11, 1.62)	0.44	0.087	0.99 (0.02, 1.95)	0.49	0.045
Domestic victimization	0.87 (0.52, 1.23)	0.18	<0.001	0.47 (0.14, 0.79)	0.17	0.005	0.81 (0.50, 1.13)	0.16	<0.001
Polyvictimisation	1.94 (1.07, 2.80)	0.44	<0.001	1.05 (0.36, 1.75)	0.35	0.003	1.74 (0.99, 2.48)	0.38	<0.001

Note. Based on complete case data (*n*=685). Robust (Huber-White) SEs were used to address some heteroscedasticity. Confounders include child sex, child ethnicity, monthly family income, maternal education, maternal work status, maternal marital status, maternal prenatal alcohol and tobacco use, child HIV exposure status, maternal antenatal depression, and child age at the 8-year CECV assessment. *Key:* CBCL=Child Behaviour Checklist; CI=confidence interval.

Appendix 4c. Cross-sectional associations between trauma exposure score at age 8 years and mental health disorders at age 8 years

	Any disorder	
	OR (95% CI)	*p*
**Unadjusted**		
Overall violence exposure	1.11 (1.06, 1.16)	<0.001
Witnessed community violence	1.08 (1.00, 1.16)	0.058
Community victimization	1.36 (1.17, 1.57)	<0.001
Witnessed domestic violence	1.50 (1.21, 1.88)	<0.001
Domestic victimization	1.18 (1.09, 1.28)	<0.001
Polyvictimisation	1.62 (1.28, 2.07)	<0.001
**Adjusted (confounders)**		
Overall violence exposure	1.08 (1.04, 1.13)	<0.001
Witnessed community violence	1.06 (0.97, 1.16)	0.171
Community victimization	1.23 (1.05, 1.43)	0.009
Witnessed domestic violence	1.45 (1.15, 1.84)	0.002
Domestic victimization	1.15 (1.06, 1.25)	0.001
Polyvictimisation	1.41 (1.09, 1.81)	0.008
**Adjusted (confounders and CBCL total score at age 3.5 years)**		
Overall violence exposure	1.08 (1.03, 1.12)	0.002
Witnessed community violence	1.04 (0.96, 1.14)	0.337
Community victimization	1.22 (1.04, 1.42)	0.014
Witnessed domestic violence	1.41 (1.11, 1.79)	0.004
Domestic victimization	1.14 (1.05, 1.24)	0.003
Polyvictimisation	1.36 (1.06, 1.76)	0.017

Note. Based on complete case data (*n*=668). Confounders include child sex, child ethnicity, monthly family income, maternal education, maternal work status, maternal marital status, maternal prenatal alcohol and tobacco use, child HIV exposure status, maternal antenatal depression, and child age at the 8-year CECV assessment. *Key:* CBCL=Child Behaviour Checklist; CI=confidence interval; OR=odds ratio.

Appendix 4d. Longitudinal associations between trauma exposure score at age 4.5 years and mental health problems at age 8 years

	**Total CBCL Score**			**Internalizing CBCL Score**			**Externalizing CBCL Score**		
	B (95% CI)	SE	*p*	B (95% CI)	SE	*p*	B (95% CI)	SE	*p*
**Unadjusted**									
Overall violence exposure	0.47 (0.23, 0.70)	0.12	<0.001	0.45 (0.26, 0.64)	0.10	<0.001	0.38 (0.17, 0.59)	0.11	<0.001
Witnessed community violence	0.54 (0.17, 0.91)	0.19	0.004	0.52 (0.23, 0.81)	0.15	<0.001	0.48 (0.14, 0.82)	0.17	0.006
Community victimization	-1.12 (-3.09, 0.84)	1.00	0.260	-0.63 (-2.09, 0.83)	0.74	0.394	-1.25 (-2.98, 0.48)	0.88	0.158
Witnessed domestic violence	0.44 (-0.53, 1.41)	0.49	0.373	0.96 (0.18, 1.73)	0.39	0.016	0.19 (-0.65, 1.04)	0.43	0.652
Domestic victimization	1.52 (0.95, 2.08)	0.29	<0.001	1.18 (0.65, 1.70)	0.27	<0.001	1.20 (0.70, 1.71)	0.26	<0.001
Polyvictimisation	1.44 (0.47, 2.41)	0.50	0.004	1.45 (0.65, 2.25)	0.41	<0.001	1.17 (0.30, 2.04)	0.44	0.008
**Adjusted (confounders)**									
Overall violence exposure	0.27 (0.02, 0.52)	0.13	0.033	0.22 (0.02, 0.43)	0.10	0.033	0.22 (-0.01, 0.44)	0.11	0.061
Witnessed community violence	0.29 (-0.11, 0.68)	0.20	0.151	0.19 (-0.11, 0.50)	0.15	0.210	0.26 (-0.10, 0.62)	0.18	0.155
Community victimization	-1.77 (-3.77, 0.23)	1.02	0.082	-1.10 (-2.45, 0.24)	0.69	0.108	-1.74 (-3.57, 0.09)	0.93	0.062
Witnessed domestic violence	-0.31 (-1.30, 0.68)	0.50	0.540	0.22 (-0.56, 1.00)	0.40	0.574	-0.42 (-1.29, 0.45)	0.44	0.341
Domestic victimization	1.22 (0.68, 1.76)	0.27	<0.001	0.88 (0.39, 1.36)	0.25	<0.001	0.97 (0.47, 1.48)	0.26	<0.001
Polyvictimisation	0.55 (-0.53, 1.64)	0.55	0.317	0.50 (-0.36, 1.36)	0.44	0.253	0.46 (-0.50, 1.41)	0.49	0.347
**Adjusted (confounders and CBCL total score at age 3.5 years)**									
Overall violence exposure	0.23 (-0.03, 0.50)	0.13	0.083	0.19 (-0.02, 0.41)	0.11	0.078	0.18 (-0.05, 0.42)	0.12	0.124
Witnessed community violence	0.24 (-0.17, 0.64)	0.21	0.259	0.16 (-0.16, 0.47)	0.16	0.333	0.22 (-0.15, 0.59)	0.19	0.242
Community victimization	-1.87 (-3.88, 0.14)	1.02	0.068	-1.18 (-2.48, 0.13)	0.66	0.077	-1.82 (-3.70, 0.07)	0.96	0.059
Witnessed domestic violence	-0.34 (-1.33, 0.66)	0.51	0.510	0.20 (-0.58, 0.99)	0.40	0.611	-0.44 (-1.32, 0.43)	0.45	0.322
Domestic victimization	1.14 (0.58, 1.69)	0.28	<0.001	0.82 (0.31, 1.32)	0.26	0.002	0.91 (0.39, 1.43)	0.26	0.001
Polyvictimisation	0.37 (-0.72, 1.46)	0.56	0.505	0.37 (-0.50, 1.24)	0.44	0.405	0.32 (-0.64, 1.28)	0.49	0.515

Note. Based on complete case data (*n*=566). Robust (Huber-White) SEs were used to address some heteroscedasticity. Confounders include child sex, child ethnicity, monthly family income, maternal education, maternal work status, maternal marital status, maternal prenatal alcohol and tobacco use, child HIV exposure status, and maternal antenatal depression. *Key:* CBCL=Child Behaviour Checklist; CI=confidence interval; SE=standard error.

Appendix 4e. Longitudinal associations between trauma exposure score at age 4.5 years and mental health disorders at age 8 years

	**Any disorder**	
	OR (95% CI)	*p*
**Unadjusted**		
Overall violence exposure	1.01 (0.95, 1.08)	0.657
Witnessed community violence	0.99 (0.90, 1.09)	0.857
Community victimization	0.99 (0.60, 1.63)	0.978
Witnessed domestic violence	1.04 (0.80, 1.34)	0.784
Domestic victimization	1.11 (0.97, 1.27)	0.125
Polyvictimisation	1.04 (0.80, 1.35)	0.777
**Adjusted (confounders)**		
Overall violence exposure	0.99 (0.92, 1.06)	0.727
Witnessed community violence	0.95 (0.86, 1.06)	0.379
Community victimization	0.91 (0.56, 1.48)	0.698
Witnessed domestic violence	0.99 (0.75, 1.31)	0.942
Domestic victimization	1.08 (0.93, 1.24)	0.320
Polyvictimisation	0.95 (0.71, 1.27)	0.729
**Adjusted (confounders and CBCL total score at age 3.5 years)**		
Overall violence exposure	0.98 (0.91, 1.05)	0.529
Witnessed community violence	0.94 (0.85, 1.05)	0.280
Community victimization	0.90 (0.55, 1.45)	0.655
Witnessed domestic violence	0.99 (0.74, 1.31)	0.923
Domestic victimization	1.06 (0.91, 1.23)	0.466
Polyvictimisation	0.92 (0.69, 1.23)	0.563

Note. Based on complete case data (*n*=561). Confounders include child sex, child ethnicity, monthly family income, maternal education, maternal work status, maternal marital status, maternal prenatal alcohol and tobacco use, child HIV exposure status, and maternal antenatal depression. *Key:* CBCL=Child Behaviour Checklist; CI=confidence interval; OR=odds ratio.

Appendix 4f. Sex differences

We conducted sex-specific sensitivity analyses using complete case data. Eight interaction effects were significant. The longitudinal association between community victimization score at age 4.5 years and any psychiatric disorder at age 8 significantly differed by sex (interaction effect *p*-value=0.024). Sex stratified analyses revealed that the community victimization OR in girls (adjusted OR 1.91 [95% CI 0.84, 4.35], *p*=0.120) was greater than the OR for boys (0.71 [0.37, 1.35], *p*=0.299), though associations for both sexes were non-significant. All other significant interaction effects were cross-sectional models. The associations between the total violence exposure score at age 8 and both the total problems score (interaction effect *p*-value=0.042) and the externalizing problems score (interaction effect *p*-value=0.020) significantly differed by sex. Sex stratified analyses revealed that violence exposure was more strongly associated with total (0.53 [0.27, 0.78], *p*<0.001) and externalizing problem scores (0.52 [0.29, 0.74], *p*<0.001) in boys compared to girls (total: 0.22 [-0.07, 0.51], *p*=0.132; externalizing: 0.17 [-0.08, 0.41], *p*=0.190). Furthermore, the associations between witnessed domestic violence score at age 8 and all three problem scores at age 8 significantly differed by sex (total: *p*<0.001; internalizing: *p* =0.007; externalizing: *p*=0.001). Sex stratified analyses again revealed a greater impact of witnessed domestic violence on mental health problems in boys (total: 2.80 [1.34, 4.26], *p*<0.001; internalizing: 1.59 [0.32, 2.85], *p*=0.014; externalizing: 2.20 [0.81, 3.58], *p*=0.002) compared to girls (total: -1.14 [-2.66, 0.38], *p*=0.142; internalizing: -0.45 [-1.53, 0.63], *p*=0.412; externalizing: -0.77 [-2.02, 0.47], *p*=0.224). Finally, the association between the polyvictimisation score at age 8 and both the total problems score (*p*=0.005) and the externalizing problems score (*p*<0.001) significantly differed by sex. Again, sex stratified analyses revealed a greater impact of polyvictimisation on total and externalizing problem scores in boys (total: 2.92 [1.69, 4.15], *p*<0.001; externalizing: 2.91 [1.83, 3.99], *p*<0.001) compared to girls (total: 0.62 [-0.60, 1.84]), *p*=0.320; externalizing: 0.19 [-0.81, 1.19], *p*=0.710).

Appendix 5. Linear regression diagnostics

Examination of the distribution of regression model residuals using histograms consistently revealed a bimodal distribution with two distinct peaks. Panels A-F in Appendix 5b present the residual distribution for the fully adjusted cross-sectional associations between violence exposure scores at age 8 and the total problem score of the CBCL at age 8 as an example of this. We observed an unexpected peak of highly negative residuals, indicating that children were scoring substantially below their predicted CBCL score based on their CECV score.

Examination of the data (in the context of the cross-sectional model linking the total violence score to the total problem score) revealed that a number of children had scored zero on the CBCL (*n*=73 [8.9%]; *t*-scores of 24 for males and 25 for females), and that these children made up the majority of those with highly negative residuals (91 children had residuals that were less than -15; of these, 58 children had a score of zero on the CBCL [63.7%]). We posited that this pattern could reflect a) resilience (despite exposure to violence, these children have very few emotional and behaviour problems), or b) measurement error. For the latter, our primary concern was regarding the informant who completed the CBCL. To be eligible to complete the CBCL for a child, a caregiver must have been living with or caring for the child for three or more days per week. This was to ensure that the informant knew the child well enough to answer the questionnaires, though it is possible that this may not be the case even with this eligibility criteria. Unfortunately, the CBCL informant was not recorded, and we were therefore unable to investigate whether the informant was related to having a CBCL score of zero. We did examine other potential predictors of this, including maternal work status at age 8, which we posited could be a good proxy for CBCL informant (if the mother was working, it was more likely that she would not be able to complete the CBCL for her child either at the clinic or via telephone). Maternal work status at age 8 was not significantly associated with having a CBCL score of zero (OR 1.52 [0.93, 2.49], *p*=0.096) or with having a highly negative residual (1.32 [0.85, 2.06], *p*=0.216). Additionally, none of the sociodemographic variables included as confounders in our models were associated with having a CBCL score of zero at age 8 years.

Unfortunately, we were unable to definitively conclude whether these zero scores on the CBCL were due to resilience or measurement error without introducing any bias into our analyses. Given that we were unable to identify any plausible proxies for the CBCL informant, and that we had explored the data and attempted to explain this pattern of findings (as presented here), we proceeded with our analyses as planned. It is worth noting that should this higher-than-expected number of zero scores on the CBCL reflect measurement error, the estimates presented in our models (specifically those using the CBCL outcomes) may underestimate the association between violence exposure and child emotional and behavioral problems. Our violence-disorder models (using the MINI-KID outcome) may provide a more accurate and unbiased picture of this association given that diagnoses were obtained via

clinical interviews delivered to children (in the presence of a caregiver) by research assistants trained to conduct the interview.

Appendix 5b. Residual distributions for the fully adjusted cross-sectional associations between violence exposure scores and the total problem score on the CBCL at age 8 years

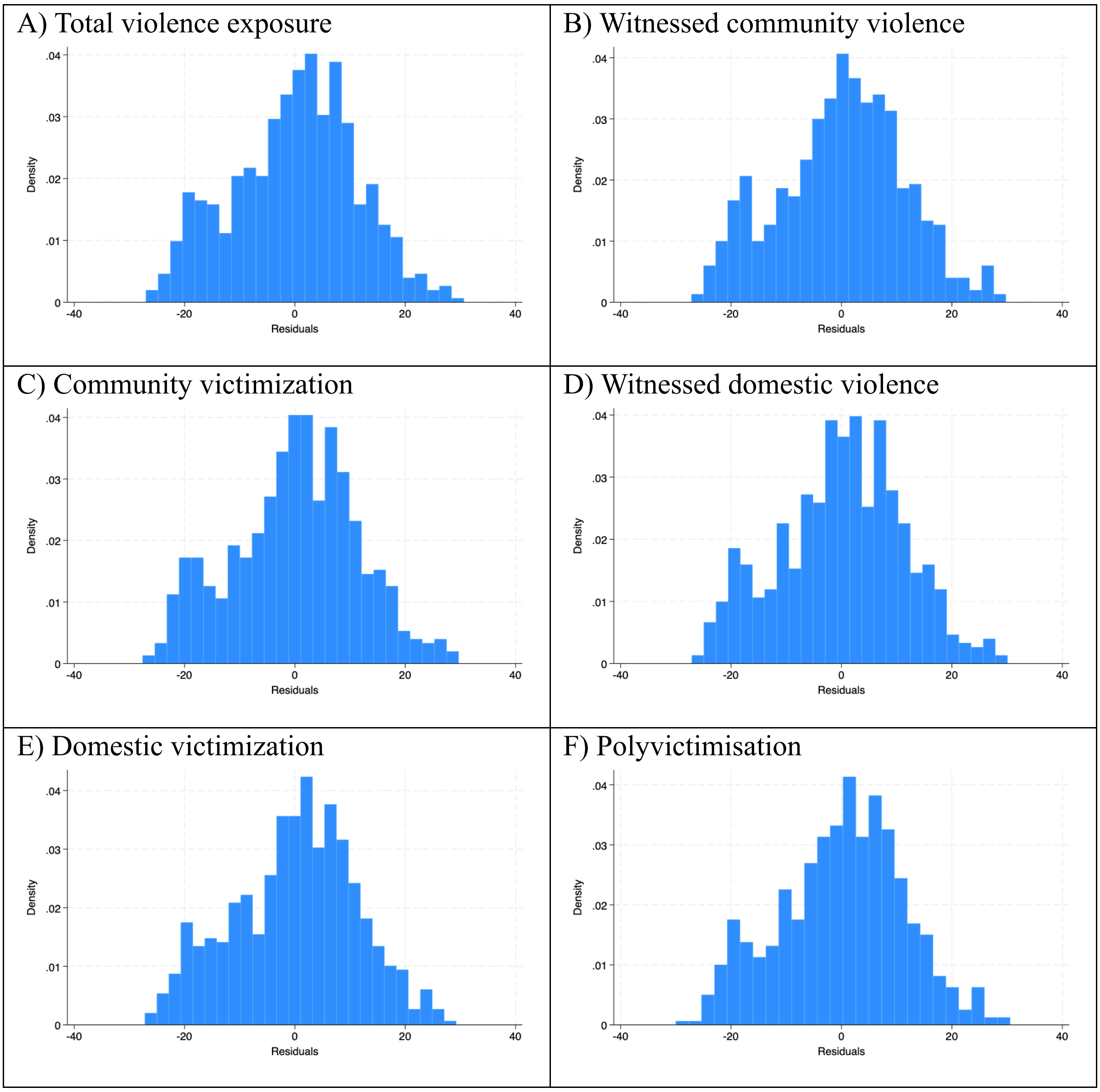

Appendix 6. Comparison of sociodemographic characteristics for children included versus excluded in the analysis sample

	**Total Sample**		**Included (*n*=974)**		**Excluded (*n*=169)**		**OR or Mean Difference (95% CI)**	***p*-value**
	**N**	***n*(%) or M(SD)**	**N**	***n*(%) or M(SD)**	**N**	***n*(%) or M(SD)**		
Female sex	1143	557 (48.7%)	974	479 (49.2%)	169	78 (46.2%)	0.89 (0.64, 1.23)	0.468
Child ethnicity (Black African)[a]	1142	632 (55.3%)	974	530 (54.4%)	168	102 (60.7%)	1.29 (0.93, 1.81)	0.130
Child HIV exposure (exposed, uninfected)[b]	1143	248 (21.7%)	974	212 (21.8%)	169	36 (21.3%)	0.97 (0.65, 1.45)	0.893
Monthly family income (R1000-5000/m)[c]	1142	596 (52.2%)	974	511 (52.5%)	168	85 (50.6%)	1.29 (0.88, 1.91)	0.195
Monthly family income (>R5000/m) [c]		160 (14.0%)		121 (12.4%)		39 (23.2%)	2.51 (1.55, 4.04)	<0.001
Maternal education (completed secondary/any tertiary)[d]	1141	448 (39.3%)	974	364 (37.4%)	167	84 (50.3%)	1.70 (1.22, 2.36)	0.002
Maternal employment status (working)[e]	1143	308 (27.0%)	974	255 (26.2%)	169	53 (31.4%)	1.29 (0.90, 1.84)	0.162
Maternal marital status (married/cohabiting)[f]	1143	462 (40.4%)	974	389 (39.9%)	169	73 (43.2%)	1.14 (0.82, 1.59)	0.426
Maternal prenatal alcohol use (yes)	1067	137 (12.8%)	933	120 (12.9%)	134	17 (12.7%)	0.98 (0.57, 1.70)	0.955
Maternal prenatal smoking (yes)	1142	323 (28.3%)	974	287 (29.5%)	168	36 (21.4%)	0.65 (0.44, 0.97)	0.034
Maternal antenatal depression	994	9.45 (5.28)	863	9.51 (5.26)	131	9.03 (5.38)	0.48 (-0.49, 1.45)	0.330

Note. [a]Reference group is mixed ancestry. [b]Reference group is HIV unexposed. [c]Reference group is <R1000/m. [d]Reference group is primary/some secondary. [e]Reference group is not working. [f]Reference group is single. *Key:* CI=confidence interval; OR=odds ratio.

Appendix 7. Prevalence of each traumatic event at the 4.5- and 8-year follow-ups

	4.5 years			8 years		
	**Total (n=745)**	**Boys (n=374)**	**Girls (n=371)**	**Total (n=836)**	**Boys (n=423)**	**Girls (n=413)**
**Any violence exposure**	**559 (75.0%)**	**286 (76.5%)**	**273 (73.6%)**	**760 (90.9%)**	**396 (93.6%)**	**364 (88.1%)**
**Witnessed community violence**	**496 (66.6%)**	**259 (69.3%)**	**237 (63.9%)**	**635 (76.0%)**	**345 (81.6%)**	**290 (70.2%)**
Heard gunshots	313 (42.0%)	179 (47.9%)	134 (36.1%)	476 (56.9%)	266 (62.9%)	210 (50.9%)
Seen someone beaten up in the neighborhood	340 (45.6%)	168 (44.9%)	172 (46.4%)	428 (51.2%)	230 (54.4%)	198 (47.9%)
Seen dead body in the neighborhood	63 (8.5%)	39 (10.4%)	24 (6.5%)	87 (10.4%)	55 (13.0%)	32 (7.8%)
Seen somebody point a gun at another in the neighborhood	41 (5.5%)	28 (7.5%)	13 (3.5%)	36 (4.3%)	27 (6.4%)	9 (2.2%)
Seen somebody get shot in the neighborhood	21 (2.8%)	15 (4.0%)	6 (1.6%)	21 (2.5%)	9 (2.1%)	12 (2.9%)
Seen somebody point a knife at another in the neighborhood	117 (15.7%)	67 (17.9%)	50 (13.5%)	121 (14.5%)	79 (18.7%)	42 (10.2%)
Seen somebody get stabbed in the neighborhood	63 (8.5%)	37 (9.9%)	26 (7.0%)	70 (8.4%)	48 (11.4%)	22 (5.3%)
Seen someone forced to do something sexual neighborhood	0 (0%)	0 (0%)	0 (0%)	1 (0.1%)	1 (0.2%)	0 (0%)
Child known someone killed by another	29 (3.9%)	19 (5.1%)	10 (2.7%)	62 (7.4%)	39 (9.2%)	23 (5.6%)
Seen someone being killed by another person elsewhere	10 (1.3%)	7 (1.9%)	3 (0.8%)	13 (1.6%)	7 (1.7%)	6 (1.5%)
**Community victimization**	**67 (9.0%)**	**39 (10.4%)**	**28 (7.6%)**	**230 (27.5%)**	**145 (34.3%)**	**85 (20.6%)**
House robbery child present	26 (3.5%)	14 (3.7%)	12 (3.2%)	45 (5.4%)	26 (6.2%)	19 (4.6%)
Someone threatened to beat up the child at school or creche	13 (1.7%)	8 (2.1%)	5 (1.4%)	102 (12.2%)	70 (16.6%)	32 (7.8%)

Someone threatened to beat up the child elsewhere	14 (1.9%)	9 (2.4%)	5 (1.4%)	55 (6.6%)	34 (8.0%)	21 (5.1%)
Child been beaten up elsewhere	21 (2.8%)	15 (4.0%)	6 (1.6%)	97 (11.6%)	63 (14.9%)	34 (8.2%)
Someone elsewhere threatened to kill the child	2 (0.3%)	0 (0%)	2 (0.5%)	4 (0.5%)	3 (0.7%)	1 (0.2%)
Someone at school or creche threatened to shoot or stab the child	0 (0%)	0 (0%)	0 (0%)	4 (0.5%)	4 (1.0%)	0 (0%)
Someone elsewhere threatened to shoot or stab the child	0 (0%)	0 (0%)	0 (0%)	2 (0.2%)	2 (0.5%)	0 (0%)
Someone shot or stabbed the child elsewhere	0 (0%)	0 (0%)	0 (0%)	0 (0%)	0 (0%)	0 (0%)
**Witnessed domestic violence**	**178 (23.9%)**	**96 (25.7%)**	**82 (22.1%)**	**204 (24.4%)**	**117 (27.7%)**	**87 (21.1%)**
Seen grownups at home hit each other	168 (22.6%)	92 (24.6%)	76 (20.5%)	180 (21.5%)	101 (23.9%)	79 (19.1%)
Seen somebody point gun at another at home	4 (0.5%)	2 (0.5%)	2 (0.5%)	9 (1.1%)	5 (1.2%)	4 (1.0%)
Seen someone at home get stabbed	21 (2.8%)	11 (2.9%)	10 (2.7%)	27 (3.2%)	20 (4.7%)	7 (1.7%)
Seen someone at home get shot	1 (0.1%)	0 (0%)	1 (0.3%)	2 (0.2%)	1 (0.2%)	1 (0.2%)
Seen someone forced to do something sexual	1 (0.1%)	1 (0.3%)	0 (0%)	0 (0%)	0 (0%)	0 (0%)
Seen someone being killed by another person at home	6 (0.8%)	2 (0.5%)	4 (1.1%)	4 (0.5%)	3 (0.7%)	1 (0.2%)
**Domestic victimization**	**168 (22.6%)**	**104 (27.8%)**	**64 (17.3%)**	**442 (52.9%)**	**234 (55.3%)**	**208 (50.4%)**
Someone threatened to beat up the child at home	14 (1.9%)	9 (2.4%)	5 (1.4%)	217 (26.0%)	111 (26.2%)	106 (25.7%)
Child been beaten up at home	15 (2.0%)	9 (2.4%)	6 (1.6%)	181 (21.7%)	88 (20.8%)	93 (22.5%)
Someone at home threatened to kill the child	2 (0.3%)	1 (0.3%)	1 (0.3%)	4 (0.5%)	3 (0.7%)	1 (0.2%)
Family member threatened to shoot or stab the child	0 (0%)	0 (0%)	0 (0%)	2 (0.2%)	2 (0.5%)	0 (0%)
Someone shot or stabbed the child at home	2 (0.3%)	2 (0.5%)	0 (0%)	0 (0%)	0 (0%)	0 (0%)

Someone made the child do something sexual	5 (0.7%)	3 (0.8%)	2 (0.5%)	10 (1.2%)	4 (1.0%)	6 (1.5%)
Family member shouts at the child fiercely and loudly	59 (7.9%)	38 (10.2%)	21 (5.7%)	67 (8.0%)	35 (8.3%)	32 (7.8%)
Anyone at home used a stick or belt or hard item to hit the child	49 (6.6%)	32 (8.6%)	17 (4.6%)	117 (14.0%)	84 (19.9%)	33 (8.0%)
Anyone at home hit the child so hard they were hurt	25 (3.4%)	13 (3.5%)	12 (3.2%)	22 (2.6%)	14 (3.3%)	8 (1.9%)
Anyone at home said the child would be sent away or kicked out	16 (2.2%)	8 (2.1%)	8 (2.2%)	75 (9.0%)	49 (11.6%)	26 (6.3%)
Anyone at home called the child horrible names	65 (8.7%)	40 (10.7%)	25 (6.7%)	116 (13.9%)	61 (14.4%)	55 (13.3%)

Note. Based on complete case data.

Appendix 8. Psychiatric disorders

Child mental health disorders were assessed at age 8 years via clinical interview using the MINI-KID [1]. Ten diagnoses were assessed: generalized anxiety disorder, obsessive compulsive disorder, posttraumatic stress disorder, separation anxiety disorder, social anxiety disorder, specific phobia, major depressive disorder, attention deficit hyperactivity disorder, conduct disorder, and oppositional defiant disorder. Few children met diagnostic criteria for each of the disorders (see Appendix 8b). We therefore were only able to examine the association between violence exposure and 'any psychiatric disorder' (binary variable capturing whether a child met criteria for at least one disorder).

Appendix 8b. Prevalence of mental health disorders at age 8 years

Diagnosis	Prevalence, *n*(%)
**Any psychiatric disorder**	**91/823 (11.1%)**
**Any internalizing disorder**	**31/836 (3.7%)**
Generalized anxiety disorder	2/838 (0.2%)
Obsessive compulsive disorder	8/853 (0.9%)
Posttraumatic stress disorder	2/846 (0.2%)
Separation anxiety disorder	2/840 (0.2%)
Social anxiety disorder	2/841 (0.2%)
Specific phobia	17/850 (2.0%)
Major depressive disorder	2/844 (0.2%)
**Any externalizing disorder**	**67/838 (8.0%)**
Attention deficit hyperactivity disorder	58/848 (6.8%)
Conduct disorder	25/843 (3.0%)
Oppositional defiant disorder	19/835 (2.3%)

Note. Based on complete case data.

Appendix 9. Sex differences

We conducted sex-specific sensitivity analyses using imputed data. Five interaction effects, all cross-sectional models, were statistically significant. The association between the total violence exposure score at age 8 and externalizing problems at age 8 significantly differed by sex (interaction effect *p*-value=0.016). Sex stratified analyses revealed a greater impact of violence exposure on externalizing problems in boys (adjusted B=0.56 [95% CI 0.36, 0.76], *p*<0.001) compared to girls (0.25 [0.03, 0.47], *p*=0.024). Furthermore, the associations between the witnessed domestic violence score at age 8 and the total problems (interaction effect *p*-value<0.001), internalizing problems (*p*=0.002), and externalizing problems scores at age 8 (*p*=0.001) all significantly differed by sex. Sex stratified analyses revealed that witnessed domestic violence was more strongly associated with total (3.01 [1.69, 4.32], *p*<0.001), internalizing (1.88 [0.68, 3.08], *p*=0.002), and externalizing problems (2.46 [1.24, 3.69], *p*<0.001) in boys but not in girls (total: -0.65 [-2.06, 0.77], *p*=0.368; internalizing: -0.32 [-1.33, 0.69], *p*=0.529; externalizing: -0.39 [-1.61, 0.84], *p*=0.535). The association between polyvictimisation at age 8 and externalizing problems at age 8 also significantly differed by sex (interaction effect *p*-value=0.020). Sex stratified analyses revealed that greater polyvictimisation was more strongly associated with externalizing scores in boys (2.11 [1.05, 3.17], *p*<0.001), but not in girls (0.58 [-0.31, 1.48], *p*=0.202).

We also found weak evidence for five further interactions: the association between the total violence score at age 8 and total problems at age 8 (interaction effect *p*-value=0.056), witnessed community violence at age 8 and both total problems (*p*=0.050) and externalizing problems at age 8 (*p*=0.078), community victimization at age 4.5 years and any psychiatric disorder at age 8 (*p*=0.083), and witnessed domestic violence at age 4.5 years and any psychiatric disorder at age 8 (*p*=0.081). In sex stratified analyses, there was a greater impact of overall violence exposure on total problem scores in boys (B=0.56 [95% CI 0.34, 0.78], *p*<0.001) compared to girls at age 8 (0.33 [0.07, 0.59], *p*=0.013). Also, there was a greater impact of witnessed community violence on total and externalizing problems in boys (total: 0.55 [0.12, 0.99], *p*=0.013; externalizing: 0.52 [0.13, 0.92], *p*=0.009) compared to girls at age 8 (total: 0.34 [-0.09, 0.77], *p*=0.119; externalizing: 0.29 [-0.06, 0.65], *p*=0.108). Surprisingly, greater community victimization by age 4.5 years was more strongly associated with meeting criteria for any psychiatric disorder at age 8 in girls (1.92 [0.93, 3.95], *p*=0.077) compared to boys (1.01 [0.66, 1.55], *p*=0.955), though both were non-significant. Similarly, greater witnessed domestic violence by 4.5 years was also more strongly associated with any

psychiatric disorder at age 8 in girls (1.40 [0.96, 2.04], p=0.078) compared to boys (0.98 [0.73, 1.31], p=0.870), though both were again non-significant.

References

1. Sheehan DV, Sheehan KH, Shytle RD, et al. Reliability and validity of the Mini International Neuropsychiatric Interview for Children and Adolescents (MINI-KID). *J Clin Psychiatry*. 2010;71(3):313-326. doi:10.4088/jcp.09m05305whi

2. Achenbach T, Rescorla L. *Manual for the ASEBA school-age forms & profiles: an integrated system of mult-informant assessment.* University of Vermont, Research Center for Children, Youth & Families; 2001.

3. Hughes RA, Heron J, Sterne JAC, Tilling K. Accounting for missing data in statistical analyses: multiple imputation is not always the answer. *International Journal of Epidemiology*. 2019;48(4):1294-1304. doi:10.1093/ije/dyz032

4. van Buuren S, Oudshoorn CGM. *Multivariate imputation by chained equations*. TNO Institute; 2000.

5. White IR, Royston P, Wood AM. Multiple imputation using chained equations: Issues and guidance for practice. *Statistics in Medicine*. 2011;30(4):377-399. doi:10.1002/sim.4067
